# HIV-related posts from a Chinese internet discussion forum: An exploratory study

**DOI:** 10.1371/journal.pone.0213066

**Published:** 2019-02-28

**Authors:** Yuan Dong, Xin Zhou, Yi Lin, Qichao Pan, Ying Wang

**Affiliations:** 1 Department of AIDS and STD, Shanghai Municipal Center for Disease Control and Prevention, Shanghai, China; 2 Department of AIDS and STD, Shanghai institutes of Preventive Medicine, Shanghai, China; Fudan University, CHINA

## Abstract

**Background:**

In China, the introduction of antiretroviral treatment has increased the number of people living with HIV/AIDS (PLWHA). New technologies, such as social media, might be useful for enhancing HIV surveillance, especially given the lack of Chinese research, which is related to stigma and discrimination. Thus, the relative anonymity of social media may make it useful for evaluating “hard to reach” PLWHA.

**Setting:**

This study used social media data to assess whether it reflected the prevalence of HIV and to explore PLWHA’ needs and online habits.

**Methods:**

In 2017, the Baidu Tieba platform was searched to obtain 2,500 HIV-related postings and 2,500 tuberculosis-related postings as a comparative sample. Word clouds and coding schemes were used to analyze the contents and review the users’ needs and online habits. Negative binomial regression was used to evaluate the relationships between word cloud geolocations and provincial numbers of men who have sex with men (MSM) PLWHA cases, after controlling for socioeconomic status.

**Results:**

Word cloud geolocations were associated with reported MSM-PLWHA cases (p<0.001). Over one-third of the HIV-related posts were seeking advice, with 40.12% being related to medical topics, although these posts received the fewest replies. The number of HIV-related social support requests was approximately 3-fold higher than the number of posts providing social support, although relatively similar proportions of support requests and support provision were observed in the tuberculosis-related postings.

**Conclusion:**

Social media may help enhance HIV surveillance. Our findings also indicate that the Chinese government, non-government organizations, and healthcare professionals should offer more online support to PLWHA.

## Introduction

According to the Chinese Center for Disease Control and Prevention, approximately 765,000 Chinese people were living with HIV/AIDS (PLWHA) by the end of 2017, which accounted for 0.05% of the total population. Furthermore, five provinces had PLWHA populations that accounted for >0.1% of the total population. The AIDS-related mortality rate in China has been continuously decreasing since the introduction of antiretroviral treatment, which suggests that HIV infection and AIDS are becoming chronic conditions in China, similar to in many developed countries[1]. The increasing longevity of affected individuals are presenting new challenges for health and social services, and a comprehensive management system has been proposed to focus on lifelong treatment and care[2]. However, data regarding PLWHA surveillance, daily habits, and daily needs are relatively limited in China[3], despite that data being important for guiding a service transition. This may be related to stigma and discrimination making it less likely for PLWHA to participate in surveillance studies[4], as these factors are prevalent in China[5]. Therefore, innovative approaches are needed to supplement existing tools and improve PLWHA surveillance systems[6].

Social media use has grown rapidly[7] and existing research has confirmed that the related data can be used in novel public health surveillance techniques[8, 9]. For example, internet discussion forums, such as Reddit, have become a popular global platform for discussion and posting questions, with specific forums (“subreddits”) that provide a virtual space dedicated to a specific topic[10]. These social websites have been recognized as a platform for efficient and inexpensive health-related communication[11], especially for “hard to reach” subpopulations, such as PLWHA[12]. This is because the forums provide a relatively anonymous environment to generate, share, and receive information with less exposure to stigma[13].

The Baidu Tieba is the largest Chinese communication platform for discussion and posting questions[14], which allows users to search or create “bars” (similar to subreddits) based on specific keywords. The HIV-related bar was created in 2007 and currently has >80,000 members, while the tuberculosis-related bar (TB-related bar) has >70,000 members. The present study collected 2,500-postings from the HIV-related bar to explore PLWHA’s needs and daily habits. Those posts were then compared to posts from the TB-related bar, which we selected based on the similar number of members, the fact that tuberculosis is a major public health problem in China[15], and the fact that patients with tuberculosis also require long-term treatment and experience discrimination. This preliminary study was designed to explore whether social media data might be used to study PLWHA, specifically regarding 1) whether geolocations from bar posts reflected reported cases of HIV infection, 2) the PLWHA’s needs, and 3) the PLWHA’s online habits.

## Materials and methods

### Data collection

The present study evaluated data from the Baidu HIV-related bar on August 29, 2017 and from the TB-related bar on August 30, 2017 respectively. Based on those dates, the 2,500 most recent posts and replies were selected for each bar. The following data were collected for each post: time of posting, username, title, content, and number of comments. After deleting duplicate posts (same contents that were posted by the same user on the same day), we obtained 2,443 HIV-related posts and 2,470 TB-related posts. Although most posts were from 2017 (2,010/2,443 for the HIV-related bar and 2,040/2,470 for the TB-related bar), we noticed that 254 posts had been created between 2005 and 2014 (103 for the HIV-related bar and 151 for the TB-related bar). Thus, to better understand PLWHA’s habits and needs during the 3 most recent years, we omitted the posts from 2005–2014 and ultimately evaluated 2,340 (144 from 2015, 186 from 2016 and 2010 from 2017) posts from the HIV-related bar and 2,319 (87 from 2015, 192 from 2016 and 2040 from 2017) posts from the TB-related bar. Socioeconomic data for each province were also collected from the Chinese National Bureau of Statistics[16], including the total population, urban population, rural population, population of web users, population with insurance, average gross domestic product, and education-related data.

In terms of ethical issues, no consent forms were sent to members of Tieba users because requesting informed consent online is difficult [17]. According to Baidu Tieba’s privacy policy, de-identified data can be used without authorization from data subjects if that data were used for academic research. (https://tieba.baidu.com/tb/cms/tieba-fe/tieba_promise.html). To minimize the potential harm to subjects, after deleting duplicate posts, the usernames of the participants were deleted. The anonymity of all the data collected was maintained throughout the study.

### Word cloud analysis

The titles of posts from both bars were extracted to separate text files, and then the jiebaR package for R software was used for Chinese text segmentation. After ignoring common Chinese stop words (e.g., “you”, “it”, “she”, and “he”) and connective words (e.g., “so”, “and”, and “thus”), we counted the frequencies of each word that appeared in the various posts. Chinese words that were repeated >10 times were subsequently translated into English (combining words with the same meaning if necessary), and then the wordcloud2 package for R software was used to create HIV-related and TB-related word clouds. Results were showed in S1 Fig.

Moreover, geographic words were counted and combined to the province level. These geolocations were used to evaluate the regional distributions of the posts.

### Category coding

Two researchers with extensive public health research experience read and categorized the posts as: (1) seeking advice, (2) sharing knowledge, (3) seeking social support (seeking emotional/ instrumental/ information support), (4) providing social support (providing emotional/ instrumental/ information support), (5) expressing emotion, or (6) others. Because some posts covered multiple categories, a single category was selected based on the predominant theme of the post. All 4,659 posts were categorized independently by the two researchers, and disagreements were resolved via discussion and consensus. A third researcher also categorized a randomly selected sample of 250 posts, and the results were used to determine the interrater reliability for each category: (1) 83.6% agreement for seeking advice, (2) 75.0% agreement for sharing knowledge, (3) 93.1% agreement for seeking social support, (4) 78.6% agreement for providing social support, (5) 92.9% agreement for expressing emotion, and (6) 86.4% agreement for others. The overall kappa value was 0.806, which is considered acceptable based on the magnitude guidelines proposed by Landis and Koch[18].

### Data analysis

Categorical variables were generally compared using the chi-square test, although Fisher’s exact test was used for expected counts of <5. Pearson correlation analysis was used to reveal correlation between the provincial-level number of reported MSM (men who have sex with men)-PLWHA cases and word cloud geolocations. Univariate and multiple negative binomial regression analyses were performed to determine the relationship between reported cases of MSM-PLWHA in 2016 and the number of regional posts per 10,000 web users in each province, after adjusting for socioeconomic characteristics. Variables with a p-value of <0.05 were fitted in a multivariate logistic regression model using a backward stepwise approach, and only factors with a significance level of <0.05 were reported. Ordinal regression was used to identify variables that were associated with popular posts, and the results were presented as odds ratios (ORs). All analyses were performed using R software (version 3.4.1), Cohen’s kappa was calculated to evaluate interrater agreement using the irr package (version 0.84) and all figures were created using the ggplot2 package (version 2.2.1).

## Results

**Fig 1:**
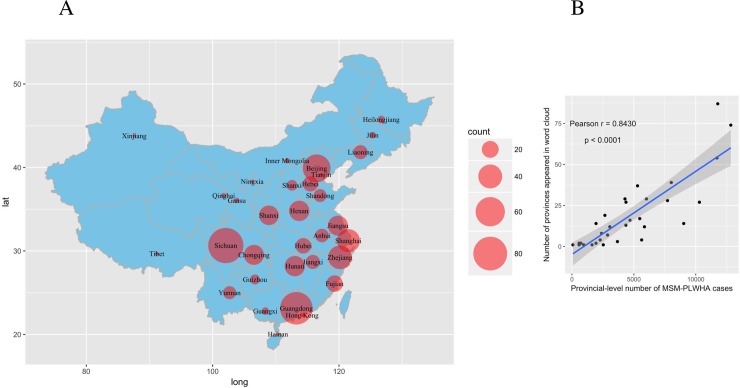
Word cloud geolocations from bar posts reflected reported cases of MSM-PLWHA. (A) The geographic distribution of the HIV-related posts, based on the number of provinces that appeared in the posts from the word cloud analysis. (B) The significant positive correlation between the MSM-PLWHA cases in 2016 and the geographic distribution of HIV-related posts (r = 0.843, p<0.0001).

Word clouds are visual representations of a body of text, with more frequently used words appearing larger in the word cloud[19], which can help rapidly summarize textual data in pictorial form. Thus, the present study used word clouds to rapidly summarize the posts, which revealed that geographic words (such as “Chengdu” and “Beijing”) appeared frequently in the HIV-related word cloud (S1A Fig). Furthermore, there were 586 HIV-related posts (25.04%) that included geographic words, relative to only 171 TB-related posts (7.37%) that included geographic words. A map was created to show the posts’ geographical distribution (Fig 1A), which is similar to the findings from a study of Chinese Weibo PLWHA users[20], who were predominantly homosexual (90.80%). Fig 1B shows a strong positive correlation between the number of province-specific geolocated HIV-related posts and the number of reported MSM-PLWHA cases in the corresponding province (r = 0.84, p<0.001). The multivariate negative binomial regression analysis also revealed a significant positive relationship between number of regional posts per 10,000 web users in each province and the province-level number of MSM-PLWHA cases, with total population and percent with a junior high school education used as the covariates (Table 1).

**Table 1:** Multivariate negative binomial regression analysis of factors associated with MSM-PLWHA cases in China, 2016.

Variables	Coefficient	Standard error	P
Provinces appearing in HIV-related posts^a^	47.1	11.9	**<0.0001**
Total population	2.1e-4	3.0e-5	**<0.0001**
Percent with a junior high school education	4.2	0.8	**<0.0001**

^a^Number of regional posts per 10,000 web users in each province.

The five most frequent words/phrases in the HIV-related bar were “HIV”, “take medicine”, “AIDS”, “find”, and “friends”. In the TB-related bar, the five most frequent words/phrases were “lung tuberculosis”, “eat”, “tuberculosis”, “take medicine”, and “medicine”. Thus, the word cloud results indicate that medication is an important concern for users of both the HIV-related and TB-related bars. The posts’ contents were subsequently analyzed based on the coding framework (S1 Table). Table 2 shows that the greatest proportion of posts in the HIV-related bar were seeking advice (n = 815, 34.83%), and that 327 posts (40.12%) were seeking advice regarding medicine. The majority of the TB-related posts were seeking advice (n = 1,358, 58.56%), including 500 posts (36.82%) that were seeking advice regarding medicine. When we analyzed the posts seeking medicine-related advice (S2 Table), we found that side effects accounted for >40% of the medicine-related posts for both the HIV-related bar (139/327, 42.51%) and the TB-related bar (249/500, 49.80%).

**Table 2:** Posting content coding results.

	HIV posts (n = 2340)		TB posts (n = 2319)		
Themes	N	%	N	%	P
**Seeking advice**					**<0.0001**
Medicine	327	40.12	500	36.82	
Tests / Clinical signs	133	16.32	372	27.39	
Study/ Work	67	8.22	87	6.41	
Hospital	40	4.91	66	4.86	
Knowledge	45	5.52	135	9.94	
High risk behavior	26	3.19	0	0	
Fertility	19	2.33	9	0.66	
Others	158	19.39	189	13.92	
Total	815	34.83	1358	58.56	
**Social support**					**<0.0001**
Seeking	559	77.0	72	41.9	
Providing	167	23.0	100	58.1	
Total	726	31.03	172	7.42	
**Expressing emotion**					**0.0030**
Positive	87	28.4	37	17.2	
Negative	219	71.6	178	82.8	
Total	306	13.08	215	9.27	
**Sharing knowledge**	**43**	**1.84**	**22**	**0.95**	
**Others**	**450**	**19.23**	**552**	**23.80**	

Seeking advice regarding tests and clinical signs was the second most common subcategory of posting for users of both the HIV-related and TB-related bars (Table 2). Among HIV-related posts seeking advice, 133 posts (16.32%) were regarding tests or clinical signs, including 74 posts (55.64%) specifically related to tests regarding CD4^+^ T-cell count (n = 27, 20.30%) and self-testing (n = 16, 12.03%), as well as 59 posts regarding clinical signs, such as sexually transmitted diseases (n = 13, 9.77%) (S3 Table). The TB-related bar included 372 posts (27.39%) regarding tests and clinical signs. The 217 posts regarding tests were main related to diagnostic imaging (144 posts, 38.71%; 111 questions regarding computed tomography, 11 questions regarding radiography, and 7 questions regarding bronchoscopy, etc.). The 155 posts regarding clinical signs included 51 posts regarding cough (13.71%).

**Fig 2:**
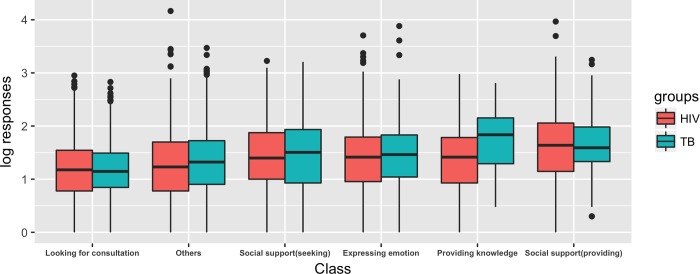
Log_10_ of posting responses. Log _10_ of the responses of different posting themes of both HIV-related bar and TB-related bar.

Interestingly, we found that posts seeking advice received the fewest responses (Fig 2), and the results from the ordinal regression (S4 Table) confirmed that posts seeking advice were less popular than even the “other” category of posts. Moreover, among posts seeking advice, the ordinal regression model revealed that questions regarding medicine and tests/clinical signs received the fewest responses (S5 Table). These results suggest that PLWHA need professional online help to address their questions regarding medicine, testing, and clinical signs.

Postings regarding social support accounted for >30% of HIV-related posts (n = 726, 31.03%) but <10% of TB-related posts (n = 172, 7.42%). Moreover, posts seeking social support were approximately 3-fold more common than posts providing social support in the HIV-related bar (559 posts vs. 167 posts), with a much more equitable distribution in the TB-related bar (72 posts vs. 100 posts). Among the 559 posts seeking social support in the HIV-related bar, the most common subcategory involved a request for friendship (n = 436, 78.00%). In the TB-related bar, only 11 of the 72 posts (15.28%) seeking social support involved a request for friendship. These results suggest that PLWHA are more likely than patients with TB to seek social support on the internet, especially emotional support.

**Fig 3:**
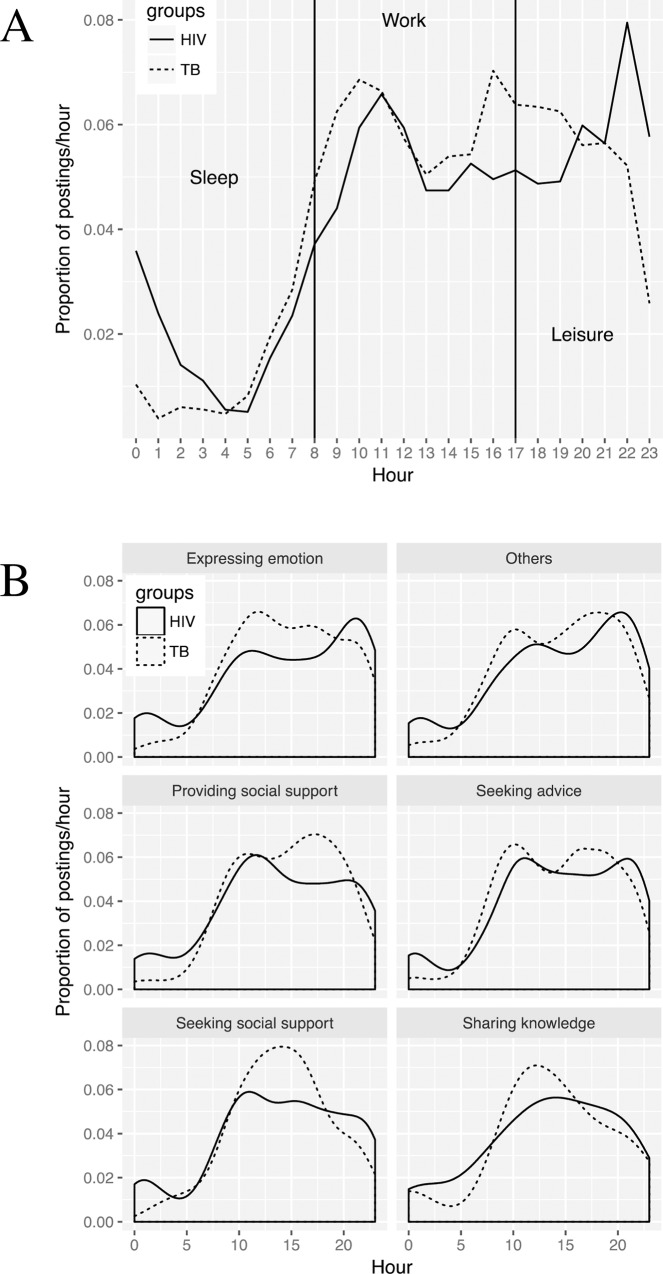
Diurnal activities of the posters from the HIV-related and TB-related Baidu Tieba forums. (A) The proportion of posts per hour related to HIV (solid line) and TB (dotted line). (B) The proportion of posts per hour stratified according to the different posting categories (indicated above each panel) related to HIV (solid line) and TB (dotted line).

The online habits of PLWHA were compared to those of the TB-related bar users based on the diurnal patterns of their postings (Fig 3A), which was evaluated as the proportion of posts made per hour. During working hours (beween 8 AM and 5 AM), the TB-related bar users seemed to be more active than the PLWHA, which may be related to the fact that patients with active TB infections cannot attend work or school. However, the PLWHA data revealed peaks late at night (between 9 PM and 4 AM). This result suggests that the TB bar users may have a relatively healthier lifestyle than PLWHA. In addition, we analyzed the hourly posting frequencies for the various coding categories. Fig 3B shows that PLWHA frequently expressed emotions late at night and were more likely to seek social support near midday. Posts by PLWHA seeking advice peaked at 10–11 AM and 9–10 PM, while posts requesting support peaked at 10–11 AM. These results indicate that there were variations in posting activity based on the purpose of the posts, which may be useful for developing schedules that allow professionals to provide online help during these peak periods.

## Discussion

Social media data may be a useful tool for predicting health outcomes[8, 9, 21, 22], and previous studies have used social media data to identify associations between risk behaviors and HIV prevalence[8]. However, to the best of our knowledge, previous studies have only included geolocated data (e.g., geolocated tweets)[8, 21, 22], despite only approximately 1% of tweets being geolocated[8] and only approximately 2% of all users providing access to their geocoded information[22]. Thus, the present study is the first use to unfiltered word cloud data, which extends the literature by exploring whether social media posts are related to disease prevalence. Our findings indicate that the word cloud geolocations in the Baidu HIV-related bar were correlated with the provincial numbers of reported MSM-PLWHA cases (r = 0.84). Interestingly, a lower yet still strong correlation (r = 0.79) was observed between the word cloud geolocations and the total PLWHA population, while a low correlation (r = 0.37) was observed between the word cloud geolocations and the non-MSM PLWHA population. This finding is consistent with previous results, which have indicated that MSM frequently use the internet to seek information[23, 24], and our data indicate that MSM may be more willing to use social media for this purpose than non-MSM PLWHA.

In China, HIV infection and AIDS are associated with greater stigma than other diseases, such as tuberculosis[25], which may prevent patients from discussing their personal needs and feelings in real life[26]. In contrast, social media offers a relatively anonymous environment for PLWHA and MSM to share information, which may explain why PLWHA tended to express their feelings and seek help using social media[27]. The present study revealed that posts regarding social support were approximately 4-fold more common in the HIV-related bar than in the TB-related bar (726 posts vs. 172 posts). Moreover, we found that HIV-related posts seeking social support were approximately 3-fold more common than posts providing social support. The imbalance between requesting and providing social support indicates that the Chinese government, non-government organizations, and healthcare professionals should offer more support to PLWHA in China.

To the best of our knowledge, this is also the first study to compare the social media data regarding HIV/AIDS to similar data regarding an unrelated chronic infectious disease. We found that users from both bars most frequently used the internet to seek advice, especially regarding medicine and tests/clinical signs. However, the ordinal regression analysis also suggested that posts seeking advice were significantly less popular than other types of posts, especially when the post was regarding medicine and tests/clinical signs. These results suggest that social media users need online help to address their questions regarding medicine, tests, and clinical signs. Moreover, we found that the HIV-related bar users frequently sought advice at 10–11 AM and 9–10 PM, which suggests that professionals who can provide online support should be available during those time periods.

This study was limited by two important factors. First, although a large and increasing number of people use social media, the resulting data are not representative of the general Chinese population. For example, one study compared internet-based and community-based samples of Chinese MSM and found that the internet-based sample was significantly younger, more educated, and more likely to accept their homosexual identity[28]. Second, the Baidu Tieba users may not be identical to the users of other online platforms, which may limit the generalizability of our findings.

Despite these limitations, the present study involved an exploratory analysis using data from the Baidu Tieba, which is the largest Chinese communication platform for posting and discussion. The results indicate that social media data may reflect the prevalence of HIV. Furthermore, the results may help both the Chinese government and health communication practitioners to improve social support for PLWHA.

## Supporting information

S1 Fig Visualization of the posts’ contents. (A) A word cloud for the postings in the HIV-related Baidu Tieba Forum. (B) A word cloud for the postings in the TB-related Baidu Tieba forum.
(DOCX)

S1 Table Outline of coding framework. (DOCX)

S2 Table Seeking medicine-related advice posts coding results. (DOCX)

S3 Table Seeking tests/clinical signs-related posts coding results. (DOCX)

S4 Table Ordinal logistic regression analysis about reply number. (DOCX)

S5 Table Ordinal logistic regression analysis about reply number of posts seeking advice. (DOCX)
