# Supplementary figures and images for: HIV-related posts from a Chinese internet discussion forum: An exploratory study
Source: PLoS One. 2019 Feb 28;14(2):e0213066. doi: 10.1371/journal.pone.0213066 (PMC6394980; doi:10.1371/journal.pone.0213066)

**
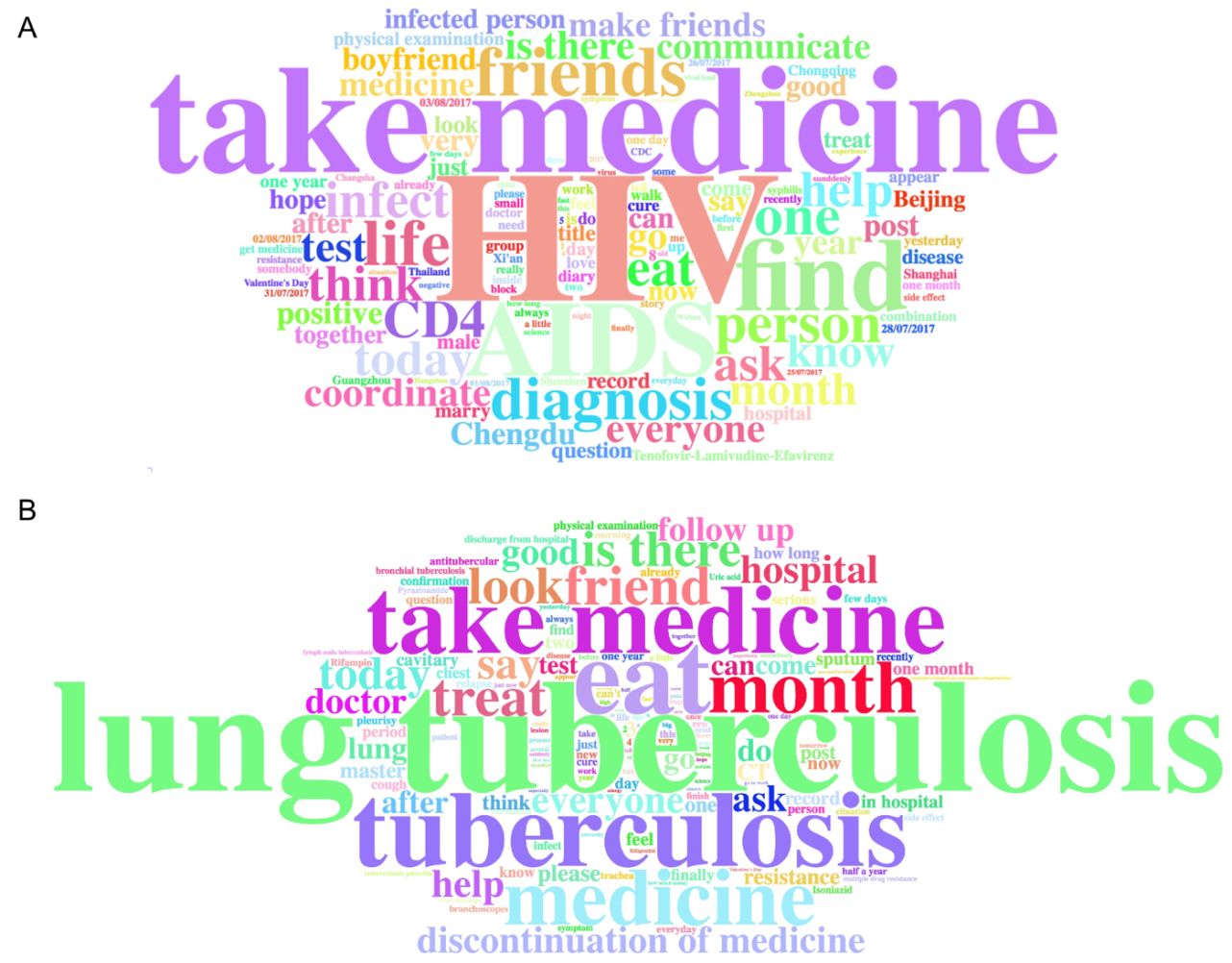
**

Supplement: S1 Fig — (A) A word cloud for the postings in the HIV-related Baidu Tieba Forum. (B) A word cloud for the postings in the TB-related Baidu Tieba forum. (DOCX) [file pone.0213066.s001.docx]
