# Supplementary material for: HIV-related posts from a Chinese internet discussion forum: An exploratory study
Source: PLoS One. 2019 Feb 28;14(2):e0213066. doi: 10.1371/journal.pone.0213066 (PMC6394980; doi:10.1371/journal.pone.0213066)
Supplement: S1 Table — (DOCX) [file pone.0213066.s002.docx]

| **Category** | **Definition** | **Example post** | **Example post(translation)** |
| --- | --- | --- | --- |
| **Expressing emotion** |  |  |  |
| Positive | Posts expressing positive emotions | 好开心。上药三个月整，cd4由378升到511，病载由30万降到小于500 | I am so happy, after taking medicine for 3 months, my CD4 count increased from 378 to 511, viral load decreased from 300,000 to less than 500 |
| Negative | Posts express negative emotions | 今天确诊了，一丝丝的希望也没有了，感觉眼前一片漆黑，好害怕，不知道未来还能走多久，担心我的父母，真不孝 | I was diagnosed today. There is no hope at all. I see completely dark in front of me, so scared. I don’t know how long I can be alive. I worry about my parent. |
| **Seeking advice** | Inquiring for information (about HIV/AIDS/TB or others) | 请问测CD4用空腹吗? | Do I need to get CD4 test done on an empty stomach? |
| **Sharing knowledge** | Posts sharing knowledge | HIV新药进展！葛兰素史克/强生每月注射一次的长效HIV疗法cabotegravir/rilpivirine进入III期临床 | New HIV treatment progress! Long-active maintenance therapy: cabotegravir/rilpivirine of GSK/ Johnson&Johnson enter phase III clinical trial |
| **Providing social support** | Providing emotional/ instrumental/ information support | 朋友们！一定坚持哦，一切都会好的！ | Friends, hang in there, everything will be fine |
| **Seeking social support** | Seeking emotional/ instrumental/ information support | 上海找个bf | I want to find a boyfriend in Shanghai |
| **Others** | Posts cannot be coded as the above categories | 早安 | Good morning |
