# Supplementary material for: HIV-related posts from a Chinese internet discussion forum: An exploratory study
Source: PLoS One. 2019 Feb 28;14(2):e0213066. doi: 10.1371/journal.pone.0213066 (PMC6394980; doi:10.1371/journal.pone.0213066)
Supplement: S2 Table — (DOCX) [file pone.0213066.s003.docx]

| **Themes** | **HIV posts(n=327)** | | | | **TB posts(n=500)** | | | |  |
| --- | --- | --- | --- | --- | --- | --- | --- | --- | --- |
|  | **N** | | **%** | | **N** | | **%** | |  |
| Side effects | | 139 | | 42.51 | | 249 | | 49.80 | |
| Normalization of medicine use | | 46 | | 14.07 | | 15 | | 10.40 | |
| Drug resistance | | 14 | | 4.28 | | 19 | | 5.40 | |
| Discontinuation of medicine | | 6 | | 1.83 | | 43 | | 8.80 | |
| Free ARV/TB medicine | | 28 | | 8.56 | | 7 | | 1.40 | |
| Treatment regimen | | 11 | | 3.36 | | 35 | | 7.40 | |
| Medicine Combinations | | 17 | | 5.20 | | 8 | | 1.60 | |
| Should I start treatment? | | 16 | | 4.89 | | 2 | | 0.40 | |
| Post-exposure prophylaxis | | 11 | | 3.36 | | 0 | | 0.00 | |
| Others | | 28 | | 1.22 | | 67 | | 1.20 | |
