# Supplementary material for: HIV-related posts from a Chinese internet discussion forum: An exploratory study
Source: PLoS One. 2019 Feb 28;14(2):e0213066. doi: 10.1371/journal.pone.0213066 (PMC6394980; doi:10.1371/journal.pone.0213066)
Supplement: S3 Table — (DOCX) [file pone.0213066.s004.docx]

|  | **HIV posts (n=133)** | |  | **TB posts (n=372)** | |
| --- | --- | --- | --- | --- | --- |
|  | **N** | **%** |  | **N** | **%** |
| **Tests** | **74** | **55.64** | **Tests** | **217** | **58.33** |
| CD4^+^T count | 27 | 20.30 | Diagnostic imaging* | 144 | 38.71 |
| Self-test | 16 | 12.03 | PPD | 26 | 6.99 |
| Viral load | 11 | 8.27 | Laboratory results | 15 | 4.03 |
| WB | 6 | 4.51 | Sputum test | 10 | 2.69 |
| Others | 14 | 10.53 | Others | 22 | 5.91 |
| **Clinical signs** | **59** | **44.36** | **Clinical signs** | **155** | **41.67** |
| Sexually transmitted diseases | 13 | 9.77 | Cough | 51 | 13.71 |
| Skin rash | 6 | 4.51 | Pain | 25 | 6.72 |
| Lymph node | 6 | 4.51 | Fever | 21 | 5.65 |
| Throat | 6 | 4.51 | Extrapulmonary tuberculosis | 20 | 5.38 |
| Others | 28 | 21.06 | Others | 38 | 10.22 |

* Diagnostic imaging posts included：111 questions regarding computed tomography, 11 questions regarding radiography, 7 questions regarding bronchoscopy, 1 question regarding b ultrasound, and 1 question regarding magnetic resonance imaging.
