# Supplementary material for: HIV-related posts from a Chinese internet discussion forum: An exploratory study
Source: PLoS One. 2019 Feb 28;14(2):e0213066. doi: 10.1371/journal.pone.0213066 (PMC6394980; doi:10.1371/journal.pone.0213066)
Supplement: S4 Table — (DOCX) [file pone.0213066.s005.docx]

|  | **No. of reply** | | | | **Univariate** | | **Multivariate** | |
| --- | --- | --- | --- | --- | --- | --- | --- | --- |
| **Variables** | **0-6** | **7-17** | **18-45** | **46-14614** | **OR** | **P** | **OR** | **P** |
| **Groups** |  |  |  |  |  |  |  |  |
| HIV | 625(26.71) | 528(22.56) | 554(23.68) | 633(27.05) | 1.00 |  | 1.00 |  |
| TB | 551(23.76) | 641(27.64) | 612(26.39) | 515(22.21) | 0.93 | 0.2247 | 1.13 | **0.0278** |
| **Time** |  |  |  |  |  |  |  |  |
| weekday | 810(24.24) | 825(24.69) | 850(25.44) | 856(25.62) | 1.00 |  | 1.00 |  |
| weekend | 366(27.77) | 344(26.1) | 316(23.98) | 292(22.15) | 0.83 | **0.0010** | 0.83 | **0.0014** |
| **Theme** |  |  |  |  |  |  |  |  |
| Others | 289(28.84) | 218(21.76) | 238(23.75) | 257(25.65) | 1.00 |  | 1.00 |  |
| Expressing emotion | 95(18.23) | 116(22.26) | 142(27.26) | 168(32.25) | 1.56 | **<0.0001** | 1.59 | **<0.0001** |
| Seeking advice | 597(27.47) | 652(30) | 561(25.82) | 363(16.71) | 0.80 | **0.0014** | 0.98 | **0.0099** |
| Sharing knowledge | 11(16.92) | 8(12.31) | 20(30.77) | 26(40) | 2.24 | **0.0006** | 2.27 | **0.0047** |
| Providing social support | 46(17.23) | 34(12.73) | 69(25.84) | 118(44.19) | 2.43 | **<0.0001** | 2.47 | **<0.0001** |
| Seeking social support | 138(21.87) | 141(22.35) | 136(21.55) | 216(34.23) | 1.45 | **<0.0001** | 1.55 | **<0.0001** |
