# Supplementary material for: HIV-related posts from a Chinese internet discussion forum: An exploratory study
Source: PLoS One. 2019 Feb 28;14(2):e0213066. doi: 10.1371/journal.pone.0213066 (PMC6394980; doi:10.1371/journal.pone.0213066)
Supplement: S5 Table — (DOCX) [file pone.0213066.s006.docx]

|  | **No. of reply** | | | | | **Univariate** | | **Multivariate** | |
| --- | --- | --- | --- | --- | --- | --- | --- | --- | --- |
| **Variables** | | **0-6** | **7-17** | **18-45** | **46-14614** | **OR** | **P** | **OR** | **P** |
| **Groups** | |  |  |  |  |  |  |  |  |
| HIV | | 234(28.71) | 223(27.36) | 206(25.28) | 152(18.65) | 1.00 |  |  |  |
| TB | | 363(26.73) | 429(31.59) | 355(26.14) | 211(15.54) | 0.95 | 0.5574 |  |  |
| **Time** | |  |  |  |  |  |  |  |  |
| Weekday | | 404(26.12) | 458(29.61) | 419(27.08) | 266(17.19) | 1.00 |  | 1.00 |  |
| Weekend | | 193(30.83) | 194(30.99) | 142(22.68) | 97(15.50) | 0.80 | **0.0094** | 0.81 | **0.0128** |
| **Theme** | |  |  |  |  |  |  |  |  |
| Others | | 89(23.73) | 90(24.00) | 100(26.67) | 96(25.60) | 1.00 |  | 1.00 |  |
| Tests/ clinical signs | | 149(29.50) | 150(29.70) | 132(26.14) | 74(14.65) | 0.61 | **<0.0001** | 0.62 | **0.0001** |
| Medicine | | 234(28.30) | 271(32.77) | 211(25.51) | 111(13.42) | 0.60 | **<0.0001** | 0.60 | **<0.0001** |
| High risk behavior | | 10(38.46) | 7(26.92) | 2(7.69) | 7(26.92) | 0.53 | 0.1077 | 0.52 | 0.1010 |
| Hospital | | 32(30.19) | 36(33.96) | 24(22.64) | 14(13.21) | 0.55 | **0.0022** | 0.54 | **0.0022** |
| Knowledge | | 58(32.22) | 48(26.67) | 51(28.33) | 23(12.78) | 0.57 | **0.0007** | 0.57 | **0.0007** |
| Study/work | | 25(16.23) | 50(32.47) | 41(26.62) | 38(24.68) | 1.08 | 0.6384 | 1.08 | 0.6504 |
